# Analysis of surgical treatment of cervical spondylotic amyotrophy

**DOI:** 10.3389/fsurg.2024.1409283

**Published:** 2024-06-13

**Authors:** Zhong Yu, Haofuzi Zhang, Yanjun Wang

**Affiliations:** ^1^ Department of Emergency, Honghui Hospital, Xi'an Jiaotong Uinversity, Xi'an, China; ^2^ Department of Neurosurgery, Xijing Hospital, Fourth Military Medical University, Xi’an, China

**Keywords:** amyotrophy, cervical spondylotic, cervical vertebra, spinal disease, surgery

## Abstract

**Background:**

Cervical spondylotic amyotrophy (CSA) is a special type of cervical spondylosis based on cervical degeneration, which is mainly manifested by weakness and atrophy of upper limb muscles without obvious sensory impairment. Various diagnostic and treatment strategies used; however, discrepancies exist. We tried to discuss diagnosing and treating CSA.

**Methods:**

15 patients with CSA were diagnosed in the Orthopedics Department of the First Affiliated Hospital of Zhengzhou University, aged 42–70 years old. The duration of preoperative symptoms of amyotrophy was 6 to 240 months. 12 patients received surgical treatment, and 3 patients received conservative treatment. The patients were divided into two groups according to the site of preoperative amyotrophy. The manual muscle test was used to evaluate the patients' muscle strength pre-and postoperatively.

**Results:**

During postoperative follow-up, the muscle strength of 12 patients improved to different degrees compared to before surgery. The improvement effect was excellent in 2 cases, good in 6, and moderate in 4. There was no decrease in postoperative muscle strength compared with that before surgery. The satisfaction rate of the effect was 66.7%. The two groups had no statistically significant difference in preoperative muscle strength. The postoperative muscle strength of the proximal group was significantly better than that of the distal group.

**Conclusion:**

The surgical effect of CSA of the proximal type is significantly better than that of the distal type. The recovery effect of amyotrophy after surgery for distal type CSA is poor; thus, surgical treatment should be carefully considered.

## 1 Introduction

Cervical spondylotic amyotrophy (CSA) is a special type of cervical spondylosis based on cervical degeneration, which is mainly manifested by weakness and atrophy of upper limb muscles without obvious sensory impairment (1). In 1965, Keegan (2) first proposed this concept in an anatomical case report. According to the location of amyotrophy, it can be divided into two subtypes: proximal type (such as scapular muscle, deltoid muscle, biceps brachii) and distal type (such as triceps brachii, forearm muscle and intrinsic hand muscle) (3). The incidence rate of the disease is low, easy to misdiagnosis. At present, there are disputes about the choice of treatment methods and timing of CSA. Inui et al. believe that CSA can achieve good curative effect through conservative treatment (4). Taychi et al. suggested that those who failed conservative treatment should undergo surgical treatment (5). However, some scholars believe that CSA needs timely surgical treatment (2, 6). This study reviewed the diagnosis and treatment of 15 patients with CSA, and analyzed their clinical characteristics, diagnosis, treatment methods and efficacy.

## 2 Materials and methods

### 2.1 Inclusion and exclusion criteria

Inclusion criteria: Upper limb amyotrophy; No obvious sensory disturbance; Complete preoperative imaging examination [including cervical x-ray, computed tomography (CT), magnetic resonance imaging (MRI), and electromyogram (EMG)]; Complete regular outpatient follow-up.

Exclusion criteria: Motor neuron disease; Hirayama disease; Amyotrophic lateral sclerosis; Shoulder sleeve injury; Lost to follow-up.

### 2.2 General information

From March 2021 to June 2022, 15 patients were admitted to the Department of Orthopedics of the First Affiliated Hospital of Zhengzhou University and diagnosed with CSA, including 7 males and 8 females, aged from 42 to 70 years (average 55.1 years). The duration of preoperative amyotrophy symptoms was 6 to 240 months (average 63.6 months). The patients were divided into two groups according to the site of preoperative amyotrophy: Proximal type group, 6 cases in total; Distal type group, 6 cases in total; Another 3 cases were treated conservatively. There was no statistical significance between the two groups in age, course of the disease, and follow-up time after surgery, as shown in Table 1.

**Table 1:** Comparison of baseline data between two groups of patients.

Group type	Cases	Age (year)	Disease duration (month)	Follow-up time (month)
Proximal	6	54.33 ± 9.852	78.83 ± 84.348	8.17 ± 3.061
Distal	6	60.33 ± 6.154	32.50 ± 22.323	7.83 ± 3.125
*t* value		−1.256	1.301	0.187
*P* value		−0.234	0.223	0.856

### 2.3 Surgical methods

When patients receive conservative treatment for at least one month which is ineffective in the end, the patients shall be treated with anterior cervical decompression and fusion or posterior cervical laminoplasty according to the compression position of the lesion, the number of the lesion segments, the curvature of the cervical spine. The principle is to relieve the compression of nerve root and spinal cord and reconstruct the physiological curvature of cervical spine.

### 2.4 Follow up and efficacy evaluation

All patients were followed up at postoperative 3, 6, and 12 months and every year after that. The follow-up aimed to evaluate the recovery of muscle strength compared with before surgery, the improvement of amyotrophy after surgery, and conduct imaging review.

### 2.5 Determination of muscle strength level

The muscle strength level is divided into 6 levels: 0, 1, 2, 3, 4, and 5. The muscle strength level of 5 is normal. The muscle strength of patients before and during the follow-up was evaluated by Manual Muscle Testing (MMT), which is based on the function of the subject's muscles or muscle groups, placing the patient in different examination positions, instructing the patient to perform certain movements in a state of anti-gravity, or resistance, and achieving the maximum range of motion, which then were divided into four groups (“excellent”, “good”, “moderate”, and “bad”). It is “excellent” that the muscle strength improve by ≥2 grades than that before operation or to restore the muscle strength to the normal level after operation. The muscle strength at follow-up was better than that before operation; It is “good” that the muscle strength improve by >1 grades than that before operation; It is “moderate” that the muscle strength has no obvious improvement compared with that before operation; It is “bad” that the muscle strength decrease compared with preoperative. At the follow-up, the “excellent” or “good” group were defined as satisfactory efficacy.

### 2.6 Statistical analysis

The data were analyzed by SPSS (version 26.0) statistical software. The measurement data were expressed by mean ± standard deviation (X ± S), and the independent sample *t*-test was used to compare the two groups. The data that did not meet the normal distribution conditions were analyzed using Mann–Whitney Testing. *P* < 0.05 is statistically significant.

## 3 Results

Three of the 12 patients who received surgical treatment found high signal area on cervical MRI T2 image. 8 patients underwent anterior cervical decompression and fusion, 4 patients underwent posterior laminoplasty, and 3 patients refused surgical treatment. All 15 patients were followed up completely, with an average follow-up time of 8.2 months. All patients did not develop amyotrophy, and the muscle strength of 3 patients without surgery did not change significantly. There was no looseness or displacement of internal fixation during the follow-up period, as shown in Table 2.

**Table 2:** Clinical data of 15 patients with CSA.

No.	Age (year)	Sex	Disease duration (month)	Type^a^	T2 high signal	Muscle strength	Treatment^b^	Follow-up time (month)	Muscle strength in follow-up
1	42	M	240	P	+	2	ACDF	10	4
2	35	M	6	D	+	3	NO	5	3
3	53	M	100	P	−	2	ACDF	9	2
4	61	M	168	P	+	2	NO	5	2
5	43	M	44	P	−	3	NO	9	3
6	54	M	48	P	+	2	ACDF	8	5
7	58	M	12	D	−	3	ACDF	4	3
8	47	F	30	P	−	3	ACDF	12	4
9	70	F	80	P	−	2	ACDF	11	3
10	58	F	60	D	−	2	LP	10	2
11	62	F	48	D	−	2	LP	11	3
12	64	F	6	D	+	2	ACDF	4	3
13	51	F	48	D	−	1	LP	10	1
14	69	F	21	D	−	1	LP	8	2
15	60	F	44	P	−	3	ACDF	7	4

^a^ P, proximal type; D, distal type.

^b^ ACDF, anterior cervical decompression and fusion; LP, laminoplasty; NO, conservative treatment.

During postoperative follow-up, the muscle strength of 12 patients improved to different degrees compared with that before operation. The improvement effect was excellent in 2 cases, good in 6 cases, and moderate in 4 cases. There was no decrease in muscle strength after operation compared with that before operation. The satisfaction rate of the effect was 66.7% (8/12), as shown in Table 3. There was no statistical significance in preoperative muscle strength between the two groups (*P* = 0.310). The postoperative muscle strength of the proximal group was significantly better than that of the distal group, with a statistical difference (*P* = 0.041).

**Table 3:** Postoperative muscle strength improvement in both groups.

Type	Excellent	Good	Moderate	Bad	Satisfaction rate (%)
Proximal (*n*)	2	3	1	0	83.3
Distal (*n*)	0	3	3	0	50.0
Total (*n*)	2	6	4	0	66.6

**Figure 1:**
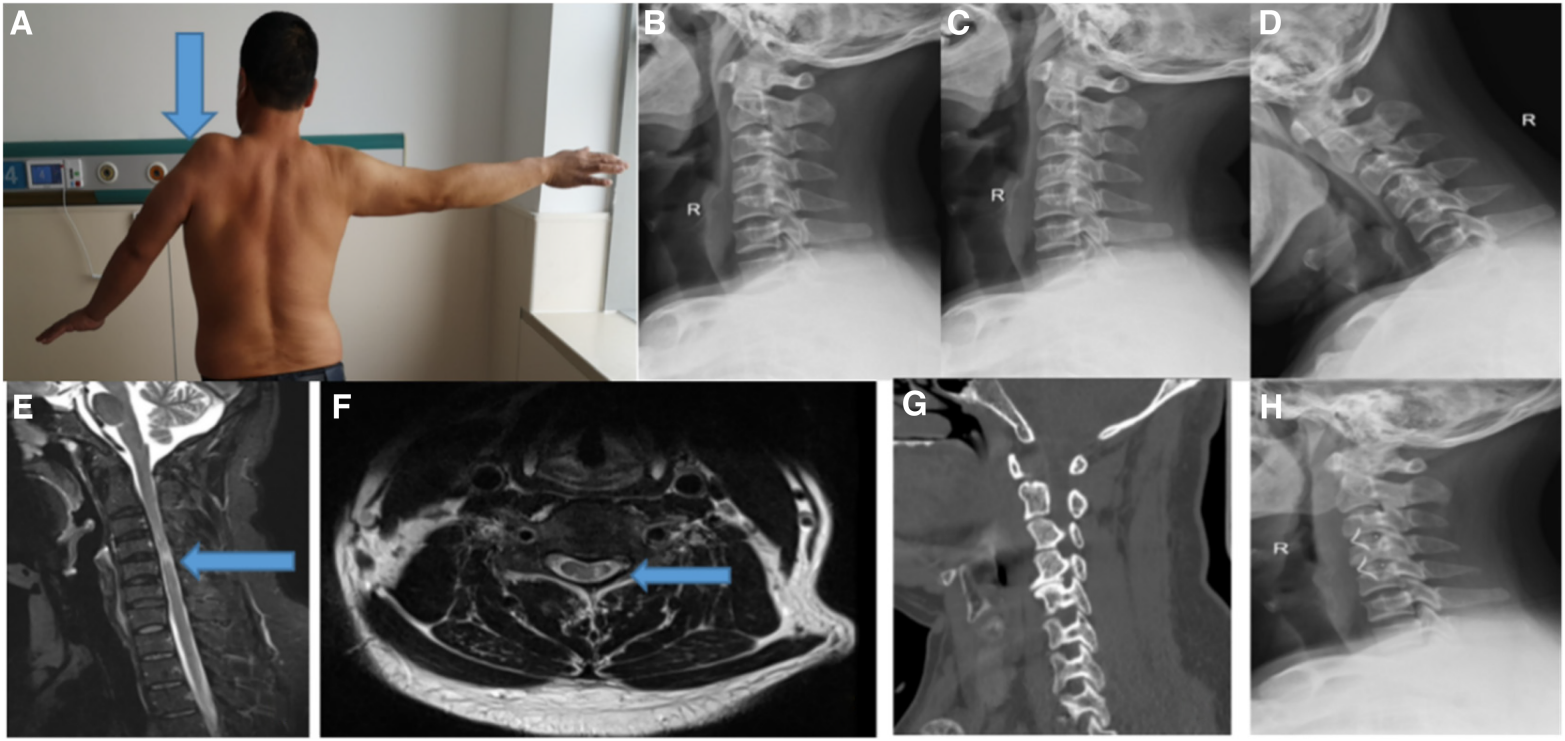
A 42-year-old male patient with proximal CSA underwent anterior cervical decompression and fusion (C3/4, C4/5). (**A**) The image before operation showed that the volume of the deltoid muscle in the left upper limb of the patient was significantly smaller than that of the right side, and the amyotrophy was obvious; (**B**) the lateral x-ray film of cervical vertebra showed that the curvature of cervical vertebra became straight before operation; (**C**) the x-ray film of the cervical spine in the extension position shows that the curvature of the cervical spine becomes straight; (**D**) x-ray film of cervical spine in anteflexion position before operation showed that the curvature of cervical spine was acceptable; (**E**) the sagittal MRI T2WI of the cervical spine showed compression of the spinal cord at C4/5 level and high signal in the spinal cord; (**F**) preoperative axial MRI showed that the left anterior horn of the spinal cord and the left nerve root were significantly compressed at C4/5 level, and the high signal changes of the bilateral anterior horn of the spinal cord, namely “snake eye sign”; (**G**) preoperative sagittal CT showed C4/5 vertebral osteophyte formation and uncinate joint hyperplasia; (**H**) three months after operation, the lateral x-ray film of cervical spine showed that the internal fixation position was good.

Typical case: a 42-year-old male patient was admitted to the hospital with “left shoulder weakness for 20 years, aggravation and left shoulder amyotrophy for 6 years”. Physical examination: The gait was stable, and the physiological curvature of the cervical spine was straightened. The movement of the cervical spine was slightly limited, with no tenderness. The left deltoid muscle was obviously atrophic with muscle strength of grade 2, and the left shoulder joint was limited in abduction. See Figure 1 for imaging data. The electromyogram (EMG) showed that the anterior horn of the spinal cord had peripheral damage. It is diagnosed as proximal CSA. Anterior cervical decompression and fusion (C3/4, C4/5) were performed, and the left deltoid muscle strength recovered to level 4 at the follow-up of 10 months after the operation.

## 4 Discussion

CSA is relatively rare in clinical practice. Keegan first proposed this concept in 1965 (2). The majority of patients are middle-aged and elderly people, and the majority of patients are unilateral, with more males than females.

### 4.1 Pathogenesis of CSA

CSA is rare in clinic, and its pathogenesis is not very clear. According to domestic and foreign literature reports, CSA may have the following types: Selective ventral spinal cord nerve root (VNR) injury: Keegan et al. believe that anterior spinal root compression is one of the important mechanisms of the disease (2); Anterior horn (AH) cell injury of spinal cord: It is reported in the literature that there is symmetrical high signal in the AH area on the MRI T2 image of some patients with CSA, which is called “snake eye sign” (7); Anterior spinal artery (ASA) blood supply insufficiency: AH mainly receives the blood supply from the terminal branch of the anterior spinal artery. On the basis of cervical degeneration, cervical instability, and cervical spinal stenosis, cervical flexion and extension activities will cause compression or traction of blood vessels inside and outside the spinal cord, thus affecting the blood supply in the spinal cord (8). The blockage of ASA blood flow causes insufficient AH perfusion in multiple segments, which is considered to be one of the pathogenesis of CSA (9); Both VNR and AH are involved: Imajo believes that the herniated intervertebral disc or osteophyte at the posterior edge of the vertebral body can cause VNR and AH to be involved at the same time (10). Most of the patients in our research group conformed to the above view, and 5 patients had “snake eye sign” on imaging. In addition, the incidence rate of CSA is not significantly related to the degree of cervical degeneration (11).

### 4.2 Diagnosis and differential diagnosis

Diagnostic criteria: Patients with cervical spondylosis or previous history of cervical spondylosis, unilateral and a few bilateral upper limb amyotrophy, and the muscle strength of the affected limb has been weakened to a certain extent, without lower limb sensory disturbance; With or without upper limb root pain, sensory disturbance and positive pathological signs; Compression segment MRI T2WI weighted image with or without high signal of spinal cord; Neuroelectrophysiological examination was consistent with cervical spondylosis of amyotrophic type; Motor neuron disease, Hirayama disease and peripheral neuropathy (such as cubital tunnel syndrome) were excluded (12).

Motor neuron disease is the most critical disease to differentiate from CSA, and amyotrophic lateral sclerosis (ALS) is the most common one (13). The main clinical manifestations are progressive aggravation of skeletal muscle weakness, atrophy, muscle bundle fibrillation, bulbar paralysis and pyramidal bundle sign, which are common in the elderly. Neuroelectrophysiological examination is very important in the differential diagnosis of the two. Repetitive nerve stimulation is mainly used to evaluate the electrophysiological test of neuromuscular junction function, which has more obvious changes in the detection of ALS patients (14). EMG can identify the cause of muscular atrophy and determine the part and degree of nerve damage. The involvement of sternocleidomastoid muscle (accessory nerve innervation) on EMG is the most important identification point between CSA and ALS (15). If it is difficult to distinguish motor neuron disease from CSA, surgery should not be performed, because surgery may aggravate the condition of motor neuron disease.

Hirayama disease, also known as atrophy of the distal part of the upper limb in young people, occurs in adolescence and is mainly male. Its clinical features include major unilateral muscle weakness and atrophy of the hand and forearm. The onset of the disease is concealed, the progress is slow, and it often recovers within a few years. Cervical magnetic resonance imaging and neuroelectrophysiological examination are helpful to differentiate Hirayama disease from CSA, and the age of onset is also one of the differences between the two (16). In addition, CSA should be differentiated from cubital tunnel syndrome and rotator cuff disease (3).

### 4.3 Treatment

At present, there is no gold standard for the treatment of CSA. Conservative treatment is the early intervention for CSA by most clinicians, especially in the early stage of the disease, including traction, neck support fixation, hyperbaric oxygen, and taking vitamin B12 or vitamin E has also proved effective for some patients (17). Therefore, most clinicians suggest that conservative treatment should be taken as the initial treatment of CSA, especially those with the following related factors: age < 50 years old, duration of symptoms < 6 months, single-segment spinal canal stenosis, intervertebral foramen stenosis and patients who are effective for traction treatment (18).

Moreover, there is no consensus on the timing and mode of CSA surgery. Most scholars believe that once CSA is confirmed, surgical treatment can at least prevent the further development of the disease, even if it cannot effectively improve the symptoms of patients. Tauchi suggested that surgical treatment should be considered if the effect of conservative treatment is not good after 4 months of CSA symptoms. The decrease of the amplitude of the action potential of the compound muscle of the deltoid and biceps to 30%–50% of the contralateral side is also considered as the indication of surgical treatment (19).

There is no unified understanding of the choice of surgical methods. Both anterior cervical decompression and fusion and simple posterior laminoplasty have been reported in CSA (20). Uchida K believes that anterior cervical decompression and fusion is effective for most patients with CSA (6). Inui believes that posterior treatment is recommended when the segment is more than 2 with spinal canal stenosis (7).

In this study, there was no significant improvement in the symptoms of 3 patients with conservative treatment during the follow-up. The muscle strength of 12 patients was improved compared with that before operation, of which 2 cases were excellent, 6 cases were good, and 4 cases were moderate. The satisfactory rate of curative effect was 66.7% (8/12), there were 5 cases of proximal type and 3 cases of distal type. Our treatment group agreed that surgical treatment had a certain effect on patients with CSA. It is suggested that the surgical treatment of proximal CSA should be more active than that of distal CSA. Of the 12 patients in this study, 8 were treated with ACDF and 4 with LP. Most of the patients' condition improved compared with that before surgery. From the perspective of pathogenic factors, spinal cord or nerve compression is the main pathogenesis, and the main goal of surgery should be decompression (21).

Our study is a retrospective study with a short follow-up time and a small sample size, which requires a large sample of multi-center prospective further research. To sum up, the surgical effect of proximal CSA is significantly better than that of distal CSA. The proximal CSA with severe compression should be actively treated with surgery. The recovery effect of amyotrophy after distal CSA is poor. Whether to choose surgical treatment remains to be explored by spine orthopedic doctors all over the world.

## 5 Conclusions

The surgical effect of CSA of the proximal type is significantly better than that of the distal type. The recovery effect of amyotrophy after surgery for CSA of distal type is poor; thus, surgical treatment should be carefully considered.

## Data Availability

The original contributions presented in the study are included in the article/Supplementary Material, further inquiries can be directed to the corresponding author.
